# Examining the Role of Eye Movements During Conversational Listening in Noise

**DOI:** 10.3389/fpsyg.2020.00200

**Published:** 2020-02-14

**Authors:** Edin Šabić, Daniel Henning, Hunter Myüz, Audrey Morrow, Michael C. Hout, Justin A. MacDonald

**Affiliations:** Hearing Enhancement and Augmented Reality Lab, Department of Psychology, New Mexico State University, Las Cruces, NM, United States

**Keywords:** hearing impairment (HI), eye gaze behavior, oculomotor, SNR (Signal-to-Noise Ratio), auditory – visual perception

## Abstract

Speech comprehension is often thought of as an entirely auditory process, but both normal hearing and hearing-impaired individuals sometimes use visual attention to disambiguate speech, particularly when it is difficult to hear. Many studies have investigated how visual attention (or the lack thereof) impacts the perception of simple speech sounds such as isolated consonants, but there is a gap in the literature concerning visual attention during *natural* speech comprehension. This issue needs to be addressed, as individuals process sounds and words in everyday speech differently than when they are separated into individual elements with no competing sound sources or noise. Moreover, further research is needed to explore patterns of eye movements during speech comprehension – especially in the presence of noise – as such an investigation would allow us to better understand how people strategically use visual information while processing speech. To this end, we conducted an experiment to track eye-gaze behavior during a series of listening tasks as a function of the number of speakers, background noise intensity, and the presence or absence of simulated hearing impairment. Our specific aims were to discover how individuals might adapt their oculomotor behavior to compensate for the difficulty of the listening scenario, such as when listening in noisy environments or experiencing simulated hearing loss. Speech comprehension difficulty was manipulated by simulating hearing loss and varying background noise intensity. Results showed that eye movements were affected by the number of speakers, simulated hearing impairment, and the presence of noise. Further, findings showed that differing levels of signal-to-noise ratio (SNR) led to changes in eye-gaze behavior. Most notably, we found that the addition of visual information (i.e. videos vs. auditory information only) led to enhanced speech comprehension – highlighting the strategic usage of visual information during this process.

## Introduction

Many challenges can complicate conversational speech comprehension, such as the presence of background noise or an individual listener’s own baseline hearing capabilities. Unless we are in a particularly quiet environment, an ensemble of noises bombards us at all times, making accurate recognition of speech sounds a significant perceptual challenge. Listeners may compensate for difficulties in speech perception, however, by strategically changing their eye gaze behavior. Such is the case for people that have the ability to “read” lips (Gailey, 1987; Altieri, 2011). Even in the absence of true lip reading, however, a listener may be able to disambiguate what they heard by looking for cues from the speaker’s mouth or face as they speak. In noisy environments in particular, an individual might glance more at the speaker’s mouth in order to gain visual cues to help in understanding speech (Aparicio et al., 2017).

Eye-gaze has long been identified as an accurate indicator of directional visual attention (Frischen et al., 2007; Hout et al., 2015; Just and Carpenter, 1976; Parasov and Chai, 2008; Pomper and Chait, 2017). It should not be surprising, therefore, that the accuracy of speech perception is also impacted by whether or not the listener allocates (or the degree to which they allocate) their visual attention to the speaker’s face and mouth movements. Early research by Sumby and Pollack (1954), for instance, demonstrated that participants were more easily able to understand speech in noise when they were able to see the speaker’s lip and facial movements, relative to when they were presented with auditory information only. Tiippana et al. (2004) also found that when participants were distracted from the face of a speaker during a listening task, they were worse at recognizing some speech sounds, such as/t/, compared to when they were not visually distracted. Similarly, Alsius et al. (2005) demonstrated that the McGurk effect (McGurk and MacDonald, 1976) – a perceptual illusion that occurs when a spoken sound and the visual speech component of another sound (e.g. lip movements) are artificially paired, leading to the perception of an intermediate phoneme – can be almost entirely eliminated with the addition of an unrelated, secondary visual task. Indeed, Gurler et al. (2015) found a significant correlation between time spent looking at the speaker’s mouth and how often the McGurk illusion was perceived by the listener.

Thomas and Jordan (2004) further highlighted the importance of visual information, specifically lip movements, during speech comprehension. These researchers first created video clips of an English speaker articulating brief consonant-vowel-consonant words (e.g. “map”) with only the face and neck of the speaker visible within the frame. They then created eight filtered versions of each clip, such that some videos included face movements while the speaker’s mouth remained static, some included mouth movement with no accompanying face movement, and others included subsets of the total features such as only mouth movement but no other facial features present. When participants were asked to identify what the speaker said, trials in which mouth movement was shown led to improved speech comprehension compared to when there was facial movement but the speaker’s mouth remained static. Alternatively, however, Rosenblum et al. (1996) posited that it might not necessarily be individual facial features that people attend to as they comprehend speech, but rather facial kinematics as a whole. Their research made use of a point-light technique whereby fluorescent points were strategically placed on locations of the face and speech recordings were captured in complete darkness so that only the placed lights were visible. Impressively, participants were able to comprehend speech better when they were shown the point-light representations of facial movements compared to when they were shown no visual information at all. Other researchers still have investigated which particular facial regions individuals look at during speech comprehension. Lusk and Mitchel (2016) found that when viewing videos of actors speaking an artificial language, observers spent the most time attending to the mouth, then to the eyes, and then the nose. Research has also shown that these eye movement patterns vary based on noise, such that noise levels influence the proportion of time spent looking at particular facial regions (Vatikiotis-Bateson et al., 1998; Buchan et al., 2008). In this case, as the noise intensity level increased, the proportion of fixations directed at the mouth increased linearly. Taken together, these results support the contention that visual attention can reliably influence auditory perception, and when visual attention is directed away from a speaker, speech comprehension may suffer.

While the discussion hitherto has been concerned primarily with overt visual orienting (e.g. deliberately orienting the eyes toward the location of attention), it is important to note that individuals need not shift their gaze overtly to alter the location of their attention. Rather, it is possible to mentally shift our attention without foveating (i.e. directly looking at) the region of interest in a process known as covert orienting (Posner, 1980). Research by Belopolsky and Theeuwes (2009) demonstrated that covertly attending to a cued location could influence mean saccadic reaction times. Specifically, the researchers made use of a paradigm wherein a cue presented to participants either correctly (valid cue) or incorrectly (invalid cue) indicated the location of a future target. The findings showed that valid cues led to shorter saccadic reaction times compared to invalid cues. This result, as well as other research concerning covert visual attention (Riggio and Kirsner, 1997; Tas et al., 2016), supports the idea that eye movement behavior is not always indicative of where attention is being directed.

A primary function of the human auditory attention system is to disentangle sounds of interest from less interesting or important sounds in the environment. During natural listening (i.e. speech perception in the real world), such as within a café, attending to what someone is saying to you might occur while others are simultaneously speaking all around you. In other situations, multiple speakers might be talking to you at once (e.g. a group of children all vying for your attention), making it difficult to pay attention to any individual speech stream. A good example of the ability of humans to perceptually separate some sounds from others is the “cocktail party effect” (Cherry, 1953), in which a listener is able to comprehend and focus on a target speaker’s speech in a noisy environment full of many competing speech streams. Indeed, this ability of the human auditory system to selectively attend to one auditory stream and, to a large degree, disregard others has been shown in dichotic listening tasks. Here, individuals are presented with two different auditory streams over headphones (Moray, 1959; Treisman, 1960; Treisman et al., 1974) and then are asked to attend to a message in one stream while disregarding messages in the other stream. Speech intelligibility has been shown to decrease as the number of competing speakers increases (Drullman and Bronkhorst, 2000), and the direction from which the masking and target speech are presented has also been shown to influence the segregation of speech streams (Bronkhorst, 2000; Drennan et al., 2003). Specifically, the closer two sound sources are in physical space, the more likely it is for masking to occur.

Intrinsically difficult speech comprehension tasks – such as trying to listen to a quiet speaker in a room of robust noise – are further complicated by the presence of hearing impairment. The ability to correctly comprehend speech diminishes significantly with age (Frisina, 2009; Yamasoba et al., 2013). This can be a product of both a decline in cognitive function or, quite commonly, deterioration of the physical structures within the cochlea (Pichora-Fuller, 2003). Presbycusis, otherwise known as age-related hearing loss, typically manifests after the age of 50, with approximately 10 million Americans suffering from hearing loss between the ages of 65 to 74 (Gordon-Salant, 2005; Arvin et al., 2013). Early symptoms include the loss of hearing for high frequency sounds, which leads to less clarity in the voices of women, and loss in clarity of certain consonants like/s/,/t/,/k/,/p/, and/f/(Frisina, 2009).

Hearing loss can occur as a result of many different factors, such as exposure to noise, or a genetic predisposition. However, a particularly large proportion of the hearing-impaired population suffer hearing loss from age-related factors alone. As people age it becomes increasingly difficult to focus on speech that is of interest when it is surrounded by other noises – especially other speech sounds (Taitelbaum-Swead and Fostick, 2016). While it has been observed that visual cues aid in speech perception, there is evidence that suggests that these cues are even more useful for older adults who suffer from age-related hearing loss (compared to older adults with normal hearing). Tye-Murray et al. (2007) conducted a study wherein normal and hearing-impaired older adults were asked to identify speech sounds across audio-only, video-only, and audiovisual conditions. Older adults with hearing impairment outperformed normal hearing individuals in the visual-only speech condition, suggesting they had developed lip-reading skills or a more nuanced use of available visual speech information. Indeed, other research has shown that elderly individuals often focus longer on the lips of speakers than do young adults during speech comprehension (Wendt et al., 2015).

In everyday speech, people attend to and process full sentences and interact with the semantics of the speech, but must also be sensitive to all the subtleties present in real-world communication. While much research has been conducted that illuminates how noise impacts speech perception (Cooke, 2006; Lewis et al., 2010), the degradation of speech perception in older adults (Lee, 2015; Taitelbaum-Swead and Fostick, 2016), and how visual attention is guided during hearing (Tharpe et al., 2008; Iversen et al., 2015), many studies investigating speech comprehension have only used brief isolated speech sounds as stimuli (Chen and Massaro, 2004; Andersen et al., 2009), such as plosives, or single, isolated words (Clayards et al., 2008). While this is clearly suitable for some research questions, it must be acknowledged that this type of listening is fundamentally different from listening during conversational speech. Moreover, few studies have investigated the role of visual attention during difficult speech perception, such as occurs in noisy environments or for individuals with hearing loss.

While other research studies have investigated how visual attention influences auditory perception of brief speech utterances (Tiippana et al., 2004; Buchan and Munhall, 2011), the goal of the present research was to better understand the role of visual attention in conversational speech perception. More specifically, our aim was to illuminate how individuals’ eye movement behavior is shaped by simulated hearing impairment and the presence of noise. Eye-gaze behavior during speech comprehension was analyzed across six different levels of background noise to assess how individuals might alter their eye movement behavior as a function of background noise intensity. We assessed speech comprehension through a series of listening tasks that included watching video clips and completing audio-only and audiovisual versions of the *Quick Speech In Noise* (QuickSIN; Killion et al., 2004) speech perception test. A comparison of these two QuickSIN tasks also allowed for an estimate of the contribution of visual speech information to speech perception in noise. Oculomotor behavior across both different levels of background noise and the presence of simulated hearing impairment was also investigated.

We had two main predictions for this study. First, we predicted that visual speech information would be more beneficial in disambiguating speech for individuals with simulated hearing impairment compared to those without simulated hearing impairment. Second, we predicted that oculomotor behavior would vary across noise levels, such that eye movements would stabilize – as evidenced by longer fixation durations – in more difficult listening conditions. This prediction was based on the notion that visual information would become more critical in perceptually challenging speech comprehension, and by similar findings in prior research (Vatikiotis-Bateson et al., 1998).

## Materials and Methods

### Participants

Seventy-one participants were recruited from the New Mexico State University Psychology Subject Pool and were compensated with partial course credit. This study was approved by the Institutional Review Board at New Mexico State University, and all participants gave written informed consent in accordance with the Declaration of Helsinki. Sixteen participants exhibited impaired hearing (defined as at least one pure tone threshold greater than or equal to 25 dB HL; thresholds at octaves between 250 and 8000 Hz were tested) and were eliminated from all subsequent analyses. Hearing impaired participants were removed so that our participants were on equal footing regarding their baseline level of hearing abilities and therefore would only vary as a function of the condition they were randomly assigned to. Of the remaining 55 participants, 13 were eliminated from analyss due to eye tracking calibration issues (e.g. wearing highly reflective glasses). The 42 remaining participants ranged in age from 18 to 25 years (*M* = 19.0). Twenty-six reported a female gender identity, 15 reported a male gender identity, and 1 reported another gender identity. Participants exhibited a mean better-ear pure tone threshold average (PTA) of 2.82 dB HL averaged over octave frequencies from 500 to 2000 Hz. Participant PTAs ranged from −3.3 to 10 dB HL (*Median* = 3.3 dB HL).

### Stimuli

#### Videos

Six videos consisting of single speakers and six videos including multiple speakers were downloaded from Youtube.com. Videos were clipped to have a duration of 2–2.5 min. Only videos where the camera was in a fixed position throughout the clip were chosen. For a clip to be suitable for the multiple speaker condition, it was necessary for both speakers to remain within the frame of the video for the duration of the clip. Any videos where the camera switched between speakers, as is common in interview style videos, were excluded from consideration as stimuli. Additionally, only videos with static backgrounds were used (to prevent the possibility of background distractions).

#### Audio QuickSIN

Eighteen sentences from the Quick Speech-In-Noise test (Etymotic Research, 2001 Elk Grove Village, IL) were used to evaluate speech perception in noise. These sentences include five keywords embedded in 4-speaker babble (Killion et al., 2004). Target sentences were presented at 70 dB(A) through headphones. The background babble varied in intensity such that the resulting signal-to-noise ratios (SNRs) ranged from 25 to 0 dB in 5 dB steps.

#### Multimodal QuickSIN

**FIGURE 1:**
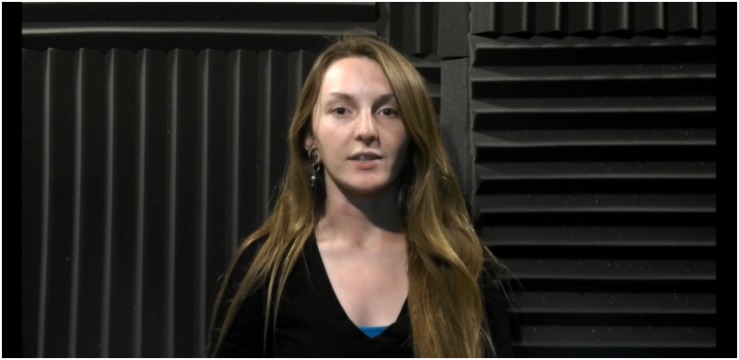
A still image from a multimodal QuickSIN trial consisting of a female speaker.

We also created a new multimodal (audio plus video) versions of the 18 QuickSIN sentences to test the contribution of visual information to speech perception (see Figure 1 for an example). The multimodal versions were comprised of video recordings of six different speakers (3 female, 3 male) reading the 18 QuickSIN sentences. All recordings were captured within the same room under identical lighting conditions, and distance between the speaker and camera was standardized via floor markings specifying both the camera and speaker locations. Recordings were made at a resolution of 1920 × 1080 pixels, and speakers gazed directly at the camera while reading the sentences in a flat affect. Each speaker was positioned so that their head and the tops of their shoulders were in the frame. The 4-speaker background speech babble was added artificially to the video recordings during post-processing to achieve SNRs ranging from 25 to 0 dB in 5 dB steps. Background speech babble was selected randomly from the 12 available speech babble tracks that accompany the QuickSIN test. Babble segments were truncated so that they had the same length as the video recordings. Ten millisecond raised cosine ramps were applied at onset and offset to the audio portion of the final stimuli. Youtube video stimuli and experiment data are available for download^1^. The QuickSIN stimuli, however, are proprietary and thus cannot be made available for download.

#### Simulated Hearing Impairment

Our experiment also included a simulated hearing impairment manipulation with two levels (normal hearing and hearing impaired). Shaped noise was added to the normal hearing stimuli to produce the hearing-impaired stimuli. We implemented the hearing impairment simulation method detailed in Desloge et al. (2010), which involves adding shaped white noise to auditory stimuli to simulate the perceptual experience of a particular listener. We simulated the hearing impairment of participant HI-1 from that paper. This participant had moderate hearing loss (pure tone detection thresholds at 15, 20, 25, 35, 40, and 35 dB HL for 250, 500, 1000, 2000, and 4000 Hz, respectively). These thresholds are associated with a Pure Tone Average threshold of 26.67 dB HL.

### Apparatus

Participants interacted with a desktop computer running the Windows 10 operating system throughout the entirety of the experiment. The experiment itself was programmed in E-prime 3.0 (Psychology Software Tools, Pittsburgh, PA, United States). A desktop-mounted Eyelink 1000 eye-tracker (SR Research, Ottawa, ON, Canada) was used to record eye movement behavior at a temporal resolution of 500 Hz. A chinrest was used throughout the experiment to maximize eye tracking accuracy. AKG K240DF circumaural headphones (AKG, Vienna, Austria) were used to present all auditory stimuli. A HP L2045w monitor with a refresh rate of 60 Hz and a resolution of 1680 × 1050 was used to display all video stimuli. The distance from the monitor to the chinrest was 30 inches. The entirety of the experiment was conducted within a sound attenuating booth (WhisperRoom, Inc., Morristown, TN, United States) to control and minimize background noise. The ambient background noise level in the sound booth was approximately 36 dB(A) measured with a sound level meter.

### Procedure

Each experiment session started by obtaining informed consent from the participant. When consent was provided, we then measured pure tone detection thresholds in both ears at octave intervals between 250 and 8000 Hz. The participant was then seated in front of the eye tracking apparatus and instructed to place their chin on the chinrest for the remainder of the experimental session. The chinrest was adjusted so each participant’s gaze landed centrally on the computer screen when the participant looked straight ahead. The participant then went through the calibration routine for the eye tracking system. The eye-tracker was then calibrated to be able to adequately track the participant’s pupil. The calibration procedure established a map of the participant’s known gaze position relative to the tracker’s coordinate estimate of that position. The routine required participants to fixate on a black circle as it moved to nine different positions (randomly) on the screen. Calibration was accepted if the mean error was less than 0.5° of visual angle, with no error exceeding 1.0° of visual angle. Periodic recalibrations ensured the accurate recording of gaze position throughout the experiment. Viewing was binocular, but only the right eye was recorded.

The participant started the main phase of the experiment once calibration was complete. Throughout the experiment, the participant interacted with the tasks via the computer keyboard, and all aspects of the experiment were self-paced. The main experiment phase started with the participant providing demographic information. The participant then proceeded to the video-viewing portion of the experiment. During each video trial, the participant was instructed to attend carefully as there would be test questions following the clip. The eye tracker was recalibrated if necessary (prior to the start of the trial) and then the participant viewed the video. Four comprehension questions (three multiple choice and one true/false) were presented after each video terminated^2^. The participant then proceeded to the next trial. The participant viewed a total of 12 videos: six single-speaker and six two-speaker. The presentation order for the 12 videos was randomized.

The audio-only QuickSIN was administered next. We utilized the standard QuickSIN procedure with the exception that responses were entered using a computer keyboard rather than verbally. At the beginning of the QuickSIN test, participants were instructed to listen for the target sentence and then type in some of the words from the sentence. A QuickSIN trial started with the presentation of the QuickSIN sentence, followed by a prompt that had the five keywords removed, e.g. “The ______ ______ ______ jumped over the ______ ______.” The participant was asked to type in the missing keywords one at a time. Keyword responses were scored as correct if they exactly matched one of the keywords in the QuickSIN stimulus. Word order was not considered during scoring, and typos/spelling errors were evaluated individually for correctness. Participants were presented with a total of three QuickSIN sentence lists (18 sentences). The number of correctly identified keywords was averaged across the three lists to produce an estimate of SNR loss.

The final phase of the experiment consisted of the multimodal version of the QuickSIN. All details of this phase were identical to the audio-only QuickSIN with the exception that multimodal stimuli were used in place of the audio-only QuickSIN stimuli, and different sentences were used to avoid practice effects. After the conclusion of the multimodal QuickSIN phase, the experiment terminated and participants were debriefed. Experiment sessions lasted approximately 90 min, including obtaining informed consent, audiometry, the main experiment, and participant debriefing.

## Results

While other research studies have investigated how different listening conditions influence the degree to which certain facial regions, such as the lips and eyes, are visually attended to Lusk and Mitchel (2016) and Vatikiotis-Bateson et al. (1998), our research question was concerned exclusively with how eye movements differ as a function of the presence or absence of simulated hearing impairment, number of speakers, and levels of signal to noise ratio. We first report analyses on the data collected during presentation of YouTube videos, and then report the analyses concerning data collected during both the audio and multimodal QuickSIN.

### Videos

**FIGURE 2:**
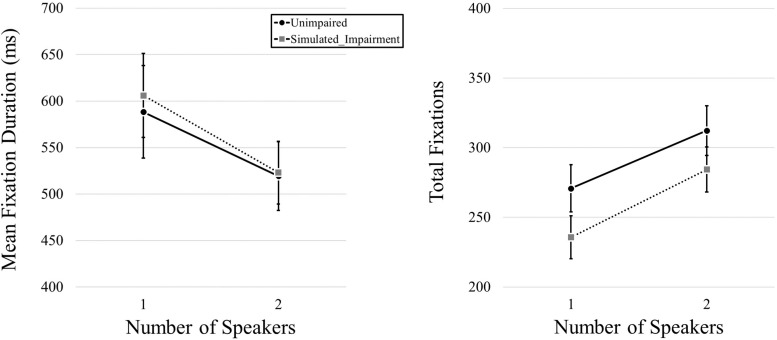
Mean fixation durations, presented as a function of the number of speakers and hearing impairment.

We began by examining fixation duration and fixation frequency as a function of simulated hearing impairment condition and the number of speakers in the video. We first conducted a MANOVA that included both mean fixation duration and total fixation frequency data as dependent variables, and simulated hearing impairment and number of speakers as independent variables. We found no effect of Simulated Hearing Impairment, *F*(2,39) = 2.615, *p* = 0.86, η*_*p*_^2^* = 0.118, but we did find an effect of Number of Speakers, *F*(2,39) = 35.885, *p* < 0.001, η*_*p*_^2^* = 0.648. The interaction was not significant, *F*(2,39) = 0.212, *p* = 0.81, η*_*p*_^2^* = 0.011. We then used a pair of 2 (Simulated Hearing Impairment: normal, simulated impairment; manipulated between participants) × 2 (Number of Speakers: 1, 2; within participants) mixed ANOVAs for follow-up analyses (one for each of the dependent variables). Here, we found no effect of Simulated Hearing Impairment on mean fixation duration, *F*(1,40) = 0.036, *p* = 0.851, but we did find an effect of Number of Speakers, *F*(1,40) = 18.238, *p* < 0.001, η*_*p*_^2^* = 0.313, with longer fixation durations for one-speaker videos (597 ms) relative to two-speaker videos (521 ms). The interaction was not significant, *F*(1,40) = 0.159, *p* = 0.692. Eye movement results for video stimuli are plotted in Figure 2. Turning to total fixation frequency data, we found no effect of Simulated Hearing Impairment, *F*(1,40) = 1.899, *p* = 0.176. There was a main effect of the Number of Speakers, *F*(1,40) = 67.614, *p* < 0.001, η*_*p*_^2^* = 0.628, with fewer fixations being committed during one-speaker videos (253.27) relative to two-speaker videos (298.40). The interaction was not significant, *F*(1,40) = 0.424, *p* = 0.519.

**FIGURE 3:**
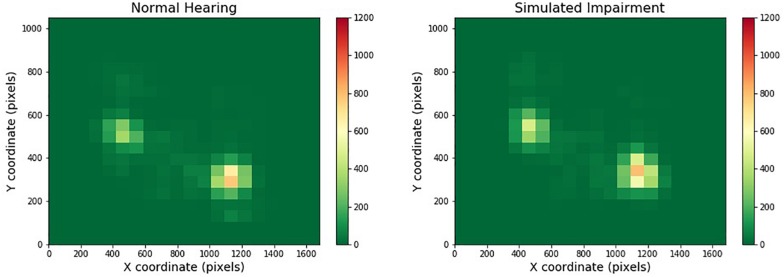
Heatmaps across one- and two-speaker videos and hearing impairment. Regions that are red or yellow indicate greater visual attention, while green regions indicate comparatively lesser visual attention.

Figure 3 depicts one of the heatmaps for our video stimuli. The heatmap shows the distribution of gaze location for normal and simulated hearing impairment groups (moving from left to right). Red cells indicate areas that were frequently gazed upon, while green areas are those that were largely ignored.

We then conducted a quantitative analysis to compare the spatial distribution of gaze across conditions. Each subject viewed a total of 12 videos, six with a single speaker and six with two speakers. For each video, the eye tracker recorded the subject’s eye fixations as (*x*, *y*) coordinates. Because the fixations varied in number, either a single bivariate normal distribution (for the single speaker videos) or a mixture of two bivariate normal distributions (for the two speaker videos) were fit to each subject’s data, separately for each video. This resulted in a vector of means for each combination of subject and video, corresponding to the mean(s) of the fitted distribution(s). One-speaker videos had a two-element vector corresponding to the *x* and *y* coordinates of the mean of the single fitted distribution, and two-speaker videos had a four-element vector corresponding to the *x* and *y* coordinates of the means of the two fitted distributions. In the two-speaker case, the mean of the leftmost fitted distribution was represented as the first two elements of the vector, and the rightmost fitted distribution was represented as the last two elements of the vector. These vectors served as the dependent variables for the subsequent Multivariate Analysis of Variance (MANOVA). Because the number of dependent variables (as well as the movies themselves) differed across the one- and two-speaker conditions, we ran two separate MANOVAs.

Starting with the one-speaker videos, we ran a 2-way mixed MANOVA with Simulated Hearing Impairment (two levels, Normal Hearing or Hearing Impaired) as a between-subjects factor and Video (six levels) as a within-subjects factor. The x and y coordinates for the mean of the fitted distribution served as dependent variables in the analysis. The Video by Simulated Hearing Impairment interaction was not significant, *F*(10,29) = 1.279, *p* = 0.287. The main effect of video was significant, *F*(10,29) = 128.529, *p* < 0.001, η*_*p*_^2^* = 0.978. The main effect of Simulated Hearing Impairment was not significant, *F*(2,37) = 1.701, *p* = 0.196.

We found very similar results with the two-speaker videos. For this analysis we ran a 2-way mixed MANOVA with Simulated Hearing Impairment (two levels, Normal Hearing or Hearing Impaired) as a between-subjects factor and Video (six levels) as a within-subjects factor. The *x* and *y* coordinates for the means of each of the fitted distributions served as dependent variables in the analysis. The Video by Simulated Hearing Impairment interaction was not significant, *F*(20,19) = 1.358, *p* = 0.254. The main effect of video was significant, *F*(20,19) = 64.082, *p* < 0.001, η*_*p*_^2^* = 0.985. The main effect of Simulated Hearing Impairment was not significant, *F*(4,35) = 1.514, *p* = 0.219. Therefore, we did not find evidence to support the idea that the spatial distribution of gaze changed with the level of hearing impairment for either one- or two-speaker videos.

### QuickSIN

To analyze the data from the QuickSIN phase of the experiment, we started by examining the eye movement data during the multimodal version of the QuickSIN test. We looked at the effects of simulated hearing impairment and Signal-to-Noise Ratio (SNR, in dB) on fixation duration and fixation frequency.

We first conducted a MANOVA that included both mean fixation duration and total fixation frequency data as dependent variables and simulated hearing impairment and SNR as independent variables. We found no effect of Simulated Hearing Impairment, *F*(2,39) = 2.760, *p* = 0.76, η*_*p*_^2^* = 0.124, but we did find an effect of SNR, *F*(10,31) = 2.556, *p* < 0.0001, η*_*p*_^2^* = 0.452. The interaction was not significant, *F*(10,31) = 0.897, *p* = 0.547, η*_*p*_^2^* = 0.224.

We then conducted a follow-up pair of 2 (Simulated Hearing Impairment) × 6 (Signal-to-Noise; 0, 5, 10, 15, 20, 25; within participants) mixed ANOVAs, one for each dependent variable. We found no effect of Simulated Hearing Impairment on fixation duration, *F*(1,40) = 0.328, *p* = 0.570. There was a main effect of SNR, *F*(1.437,57.499) = 3.918, *p* = 0.038, η*_*p*_^2^* = 0.089. As SNR increased, fixation durations decreased (980, 660, 782, 703, 703, and 694 ms for SNRs from 0 to 25 in steps of 5). The interaction was not significant, *F*(1.437,57.499) = 0.561, *p* = 0.518.

**FIGURE 4:**
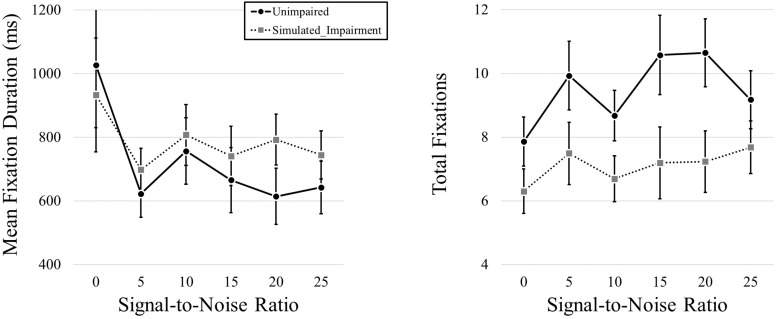
Mean fixation durations and total fixations presented across levels of SNR (measured in dB) and hearing impairment.

We found a main effect of Simulated Hearing Impairment on total fixation frequency, *F*(1,40) = 4.823, *p* = 0.034, η*_*p*_^2^* = *0.108*,with fewer fixations in the simulated hearing impairment condition (7.10), relative to the normal condition (9.48). There was also a marginally significant effect of SNR, *F*(1.752,70.060) = 3.010, *p* = 0.062, η*_*p*_^2^* = 0.070. Fixation frequency tended to increase as SNR increased (7.08, 8.71, 7.69, 8.89, 8.94, and 8.43 for SNRs from 0 - 25 in steps of 5). The interaction was not significant, *F*(1.752,70.060) = 0.998, *p* = 0.365. Eye movement results can be seen in Figure 4.

**FIGURE 5:**
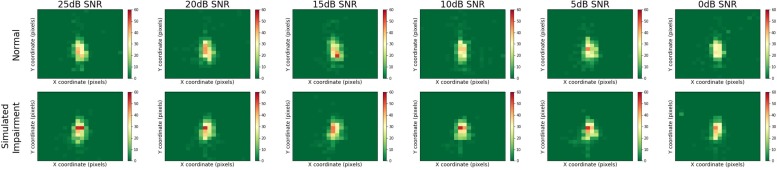
Heatmaps across SNR and hearing impairment for the multimodal QuickSIN. Regions that are red or yellow indicate greater visual attention, while green regions indicated lesser visual attention.

Figure 5 presents heatmaps for the multimodal QuickSIN stimuli, displaying results for normal and simulated hearing impairment groups down rows, and for varying levels of SNR across columns. Here, we can see that gaze location was centrally focused on the speaker of the multimodal QuickSIN video. Moreover, gaze location also seems to remain consistent across varying levels of SNR.

As with the movie data discussed above, we conducted an analysis of the spatial distribution of gaze during the multimodal QuickSIN test. In this case, Simulated Hearing Impairment was still a between-subjects factor. Signal-to-Noise Ratio (SNR) was a within-subjects factor and ranged from 25 to 0 dB in 5 dB steps. Because all speakers in the multimodal QuickSIN were located in the same position on the computer screen (unlike the movie stimuli), we combined fixation data across the videos. This resulted in a collection of gaze fixations for each combination of subject and SNR. As before, a bivariate normal distribution was fit to these data, resulting in a two-element mean vector for each combination of subject and SNR. These vectors served as the dependent variables for the MANOVA-based analysis.

We ran a 2-way mixed MANOVA with Simulated Hearing Impairment (two levels, Normal Hearing or Hearing Impaired) as a between-subjects factor and SNR (six levels) as a within-subjects factor. The x and y coordinates for the mean of the fitted distributions served as dependent variables in the analysis. The SNR by Simulated Hearing Impairment interaction was not significant, *F*(10,30) = 1.036, *p* = 0.439. The main effect of SNR was not significant, *F*(10,30) = 0.664, *p* = 0.748. The main effect of simulated hearing impairment was not significant, *F*(2,38) = 0.655, *p* = 0.525. As with the video data discussed above, we found no evidence from the multimodal QuickSIN data that the spatial distribution of gaze changes with the difficulty of the listening task (either through the simulated hearing impairment or SNR manipulations).

**FIGURE 6:**
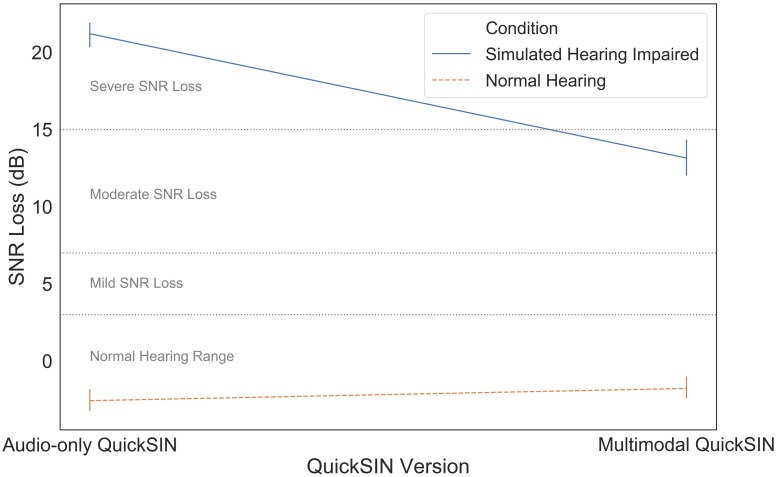
SNR loss as a function of hearing impairment and QuickSIN modality.

Finally, we compared the audio-only and multimodal versions of the QuickSIN to determine the use of visual information during different levels of simulated hearing impairment. SNR loss measures using the audio-only and multimodal QuickSIN measures were strongly correlated, *r*(40) = 0.95, *p* < 0.001. Mean SNR loss as a function of QuickSIN modality (audio-only vs. multimodal) and simulated hearing impairment (normal hearing vs. simulated hearing impaired) is illustrated in Figure 6. These data were subjected to a two-way mixed ANOVA with simulated hearing impairment as a between-subjects factor and QuickSIN modality as a within-subjects factor. There was a significant modality by simulated hearing impairment interaction, *F*(1,40) = 74.522, *p* < 0.001, η*_*p*_^2^* = 0.651. The additional visual information in the multimodal stimuli led to a 9 dB improvement in SNR loss, but only for participants in the simulated hearing impaired condition. There were also significant main effects of modality, *F*(1,40) = 43.806, *p* < 0.001, η*_*p*_^2^* = 0.523, and simulated hearing impairment *F*(1,40) = 70.143, *p* < 0.001, η*_*p*_^2^* = 0.637. The main effect of modality appears to be entirely driven by the significant modality by simulated hearing impairment interaction.

## Discussion

Our ability to process auditory environments and disentangle sounds of interest from irrelevant sounds is often taken for granted. This ability, unfortunately, degrades as we age and as a result of hearing damage. However, both normal hearing and hearing-impaired individuals may be able to make use of a number of strategies to better understand speech and other sounds in perceptually challenging conditions. The present research investigated how individuals vary their oculomotor behavior as a function of simulated hearing impairment, number of speakers in the conversation, and the degree of background noise. This research fills an apparent gap in the literature for studies that go beyond the perception of simple speech sounds, such as individual consonants, and instead makes use of either sentences or fully conversational stimuli, which include the nuances of language such as semantics.

There were three major findings in the present research: (1) visual information was especially useful for those in the simulated hearing impairment condition, (2) fixation durations were longer when participants were listening in higher levels of background noise, and (3) the spatial distribution of eye gaze was consistent across levels of simulated impairment and background noise. These results suggest that eye gaze stabilizes to some extent during difficult listening conditions, and that hearing impaired listeners may benefit especially from the presence of visual information compared to individuals with normal hearing.

The audio-only vs. multimodal QuickSIN results supported our prediction that visual speech information could be used to improve speech perception for those with simulated hearing impairment. Participants in the simulated hearing impairment condition were able to use the visual information that accompanied multimodal QuickSIN stimuli to perform significantly better than in the audio-only QuickSIN, reducing SNR loss by approximately 9 dB. It appears that participants were using the visual information provided to help capture additional information about the speech stimulus. The participants may have used cues based on lip movements to disambiguate the sentence in the presence of noise; in this case the moderate simulated hearing impairment. Interestingly, normal-hearing listeners did not show any benefit from the inclusion of this information. This points to the fact that, during challenging listening tasks such as speech comprehension in noise and in difficult SNR environments, individuals can effectively take advantage of visual information to partially counteract the effects of moderate hearing loss. This has important implications for better understanding how hearing-impaired individuals strategically change their eye gaze behavior (and listening behavior in general) to handle perceptually challenging speech comprehension. It is important to note that the audio-only QuickSIN always preceded the multimodal QuickSIN. We firmly believe that the result observed is due to our experimental manipulation and not a potential order effect for two primary reasons: (1) no QuickSIN sentences were repeated during the experiment, so participants did not have any additional information during the multimodal QuickSIN than they had during the audio-only QuickSIN (outside of the visual information), and (2) the QuickSIN is a validated diagnostic instrument that does not show practice effects as the individual’s ability to perceptually segregate noise from a target will not improve or worsen over the course of the experiment. Indeed, research has supported that repeated speech perception in noise tests are not susceptible to learning effects so long as the stimuli are not repeated (Stuart and Butler, 2014).

A significant effect of SNR on fixation duration also supported our prediction that oculomotor behavior would vary across noise levels, such that longer fixation durations would be observed under more difficult listening conditions. While eye-gaze data did not, by and large, differ in relation to simulated hearing impairment, gaze fixation duration was significantly impacted by the level of SNR. Analyses also showed a main effect of SNR on mean fixation duration, such that fixation durations generally decreased as SNR increased. In other words, more difficult listening situations appear to lead to longer fixation durations, and in general, heightened or stabilized visual attention. The implication here is that individuals rely on visual information more frequently when the level of auditory background noise is louder and more robust than the target signal, regardless of hearing impairment.

While individual distribution of gaze location did differ across one and two-speaker videos, such that there were a pair of hotspots in the two-speaker videos compared to one major hotspot region in the one-speaker heatmaps, quantitative analyses did not show any difference the gaze spatial distribution across simulated hearing impairment or noise. The video clips presented had the speakers situated in the center of the screen, where eye-gaze was most focused. This indicates that during the one-speaker videos participants were staring at the single speaker’s face for a majority of the clips, whereas during the two speaker videos, participants split their fixations between the two faces. In both situations, participants focused their gaze more on the faces rather than the surrounding environment. This behavior was consistent across levels of simulated hearing impairment and background noise. Additionally, we found that eye movement behavior varied across levels of SNR during the multimodal QuickSIN tasks. Indeed, fixations increased in duration as SNR decreased, which suggests that listeners focused longer on particular visual regions in difficult listening conditions. However, we found no evidence that listeners distribute their gaze differently according to the difficulty of the listening environment. Across all conditions in both experiments, approximately 75% of total fixation time was spent focused on the face(s) of the speaker(s).

We did, however, find an effect of simulated hearing impairment on total fixation frequency data showing that fewer fixations were observed in the simulated hearing impairment condition. Specifically, longer fixation durations were observed at lower SNRs, and a main effect of simulated hearing impairment on total fixation frequency data showed that fewer fixations were observed in the simulated hearing impairment condition. This was the only such finding, however, and the pattern of results seems to support the notion that noise is more of a consistent factor on eye movements during conversational listening than simulated hearing impairment. Taken together, the results not only suggest that individuals focus longer on critical areas of interest at lower SNRs, but also that hearing-impaired individuals tend to focus longer on particular areas of interest relative to normal-hearing individuals. While lower SNRs led to longer fixation durations across both normal and simulated hearing-impaired individuals, simulated hearing-impaired individuals needed to fixate longer on particular regions of interest to help manage the increased perceptual challenge.

These findings support the conclusion that individuals strategically adjust aspects of their oculomotor behavior in response to the level of background noise in the listening environment. In high noise environments, listeners lengthen their eye fixations and begin to make use of visual speech information to improve their speech perception. Research investigating how listeners use and direct their gaze to focus attention on a speaker is integral to better understanding the strategies people – particularly those with hearing impairment – use to understand speech.

## Conclusion

Difficulty in the perception of speech can be the result of a variety of factors, any of which can lead a person to strategically alter their listening and oculomotor behaviors. The present research gives us a better understanding of how individuals direct their gaze under different levels of hearing impairment and background noise. Importantly, this investigation incorporated conversational listening tasks and real-world noise environments to improve the generalizability of the findings. Our results showed that different levels of SNR led to unique eye movement behavior such as longer fixation durations during more difficult hearing conditions. This finding generalized to both normal hearing and simulated hearing impairment individuals. These results indicate that eye-gaze behavior may be less well predicted by hearing impairment than originally thought, and that instead, eye-gaze behavior may be more heavily impacted by the relative noise conditions in the auditory environment.

## Data Availability Statement

All raw data and multimodal video stimuli can be found here: https://osf.io/3q8y6/?view_only=89e01a3eba4c447b987f2d 3642e773c2.

## Ethics Statement

The studies involving human participants were reviewed and approved by the New Mexico State University Institutional Review Board. The patients/participants provided their written informed consent to participate in this study.

## Author Contributions

All authors contributed to the conception and design of the study and contributed to revising the manuscript either through direct edits or feedback, and read and approved the submitted version. MH and JM processed all experimental data, conducted appropriate analyses, and created the figures. EŠ, DH, MH, and JM wrote components of the manuscript. EŠ and DH led the organization of databases. EŠ was lead on drafting the work and revising it critically. All authors assisted with interpretation of the data. MH programmed the experiment. JM, EŠ, and DH wrote scripts to programmatically generate and standardize the stimuli. AM and HM substantially contributed to the acquisition of data.

## Conflict of Interest

The authors declare that the research was conducted in the absence of any commercial or financial relationships that could be construed as a potential conflict of interest.

## Footnotes

1 Videos and data can be found at https://osf.io/3q8y6/?view_only=89e01a3eba4c447b987f2d3642e773c2.

2 Experiment stimuli (including video comprehension questions) are available at https://osf.io/3q8y6/?view_only=89e01a3eba4c447b987f2d3642e773c2.

## References

[B1] Alsius A. Navarra J. Campbell R. Soto-Faraco S. (2005). Audiovisual integration of speech falters under high attention demands. *Curr. Biol.* 15 839–843. 10.1016/j.cub.2005.03.046 15886102

[B2] Altieri N. (2011). Some normative data on lip-reading skills (L). *J. Acoust. Soc. Am.* 130 1–4. 10.1121/1.3593376 21786870 PMC3155585

[B3] Andersen T. S. Tiippana K. Laarni J. Kojo I. Sams M. (2009). The role of visual spatial attention in audiovisual speech perception. *Speech Commun.* 51 184–193. 10.1016/j.specom.2008.07.004

[B4] Aparicio M. Peigneux P. Charlier B. Baleriaux D. Kavec M. Leybart J. (2017). The neural basis of speech perception through lipreading and manual cues: evidence from deaf native users of cued speech. *Front. Psychol.* 8:426. 10.3389/fpsyg.2017.00426 28424636 PMC5371603

[B5] Arvin B. Prepageran N. Raman R. (2013). “High frequency presbycusis”-is there an earlier onset? *Indian J. Otolaryngol. Head Neck Surg.* 65 480–484. 10.1007/s12070-011-0356-x 24427701 PMC3889367

[B6] Belopolsky A. V. Theeuwes J. (2009). When are attention and saccade preparation dissociated? *Psychol. Sci.* 20 1340–1347. 10.1111/j.1467-9280.2009.02445.x 19788530

[B7] Bronkhorst A. W. (2000). The cocktail party phenomenon: a review of research on speech intelligibility in multiple-talker conditions. *Acta Acust. U. Acust.* 86 117–128.

[B8] Buchan J. N. Munhall K. G. (2011). The influence of selective attention to auditory and visual speech on the integration of audiovisual speech information. *Perception* 40, 1164–1182. 10.1068/p6939 22308887

[B9] Buchan J. N. Paré M. Munhall K. G. (2008). The effect of varying talker identity and listening conditions on gaze behavior during audiovisual speech perception. *Brain Res.* 1242 162–171. 10.1016/j.brainres.2008.06.083 18621032 PMC2630206

[B10] Chen T. H. Massaro D. W. (2004). Mandarin speech perception by ear and eye follows a universal principle. *Percept. Psychophys.* 66 820–836. 10.3758/bf03194976 15495907

[B11] Cherry E. C. (1953). Some experiments on the recognition of speech, with one and with two ears. *J. Acoust. Soc. Am.* 25 975–979. 10.1121/1.1907229

[B12] Clayards M. Tanenhaus M. K. Aslin R. N. Jacobs R. A. (2008). Perception of speech reflects optimal use of probabilistic speech cues. *Cognition* 108 804–809. 10.1016/j.cognition.2008.04.004 18582855 PMC2582186

[B13] Cooke M. (2006). A glimpsing model of speech perception in noise. *J. Acoust. Soc. Am.* 119 1562–1573. 10.1121/1.2166600 16583901

[B14] Desloge J. G. Reed C. M. Braida L. D. Perez Z. D. Delhorne L. A. (2010). Speech reception by listeners with real and simulated hearing impairment: effects of continuous and interrupted noise. *J. Acoust. Soc. Am.* 128 342–359. 10.1121/1.3436522 20649229 PMC2921434

[B15] Drennan W. R. Gatehouse S. Lever C. (2003). Perceptual segregation of competing speech sounds: the role of spatial location. *J. Acoust. Soc. Am.* 114 2178–2189. 10.1121/1.1609994 14587615

[B16] Drullman R. Bronkhorst A. W. (2000). Multichannel speech intelligibility and talker recognition using monaural, binaural, and three-dimensional auditory presentation. *J. Acoust. Soc. Am.* 107 2224–2235. 10.1121/1.428503 10790048

[B17] Etymotic Research (2001). *QuickSIN Speech in Noise Test Version 1.3.*

[B18] Frischen A. Bayliss A. P. Tipper S. P. (2007). Gaze cueing of attention: visual attention, social cognition, and individual differences. *Psychol. Bull.* 133 694–724. 10.1037/0033-2909.133.4.694 17592962 PMC1950440

[B19] Frisina R. D. (2009). Age-related hearing loss. *Ann. N. Y. Acad. Sci.* 1170 708–717. 10.1111/j.1749-6632.2009.03931.x 19686217

[B20] Gailey L. (1987). “Psychological parameters of lip-reading skill,” in *Hearing by Eye: The Psychology of Lip-Reading*, eds Dodd R. Campbell B., (Hillsdale, NJ: Erlbaum), 115–141.

[B21] Gordon-Salant S. (2005). Hearing loss and aging: new research findings and clinical implications. *J. Rehabil. Res. Dev.* 42 9–24. 16470462 10.1682/jrrd.2005.01.0006

[B22] Gurler D. Doyle N. Walker E. Magnotti J. Beauchamp M. (2015). A link between individual differences in multisensory speech perception and eye movements. *Attent. Percept. Psychophys.* 77 1333–1341. 10.3758/s13414-014-0821-1 25810157 PMC4437244

[B23] Hout M. C. Walenchok S. C. Goldinger S. D. Wolfe J. M. (2015). Failures of perception in the low-prevalence effect: evidence from active and passive visual search. *J. Exp. Psychol. Hum. Percept. Perform.* 41 977–994. 10.1037/xhp0000053 25915073 PMC5543182

[B24] Iversen J. Patel A. Nicodemus B. Emmorey K. (2015). Synchronization to auditory and visual rhythms in hearing and deaf individuals. *Cognition* 134 232–244. 10.1016/j.cognition.2014.10.018 25460395 PMC4255154

[B25] Just M. A. Carpenter P. A. (1976). Eye fixations and cognitive processes. *Cogn. Psychol.* 8 441–480. 10.1016/0010-0285(76)90015-3

[B26] Killion M. C. Niquette P. A. Gudmundsen G. I. (2004). Development of a quick speech-in-noise test for measuring signal-to-noise ratio loss in normal-hearing and hearing-impaired listeners. *J. Acoust. Soc. Am.* 116 2395–2405. 10.1121/1.1784440 15532670

[B27] Lee J. (2015). Aging and speech understanding. *J. Audiol. Otol.* 19 7–13. 10.7874/jao.2015.19.1.7 26185785 PMC4491939

[B28] Lewis D. Hoover B. Choi S. Stelmachowicz P. (2010). The relationship between speech perception in noise and phonological awareness skills for children with normal hearing. *Ear Hear.* 31 761–768. 10.1097/aud.0b013e3181e5d188 20562623 PMC3358133

[B29] Lusk L. G. Mitchel A. D. (2016). Differential gaze patterns on eyes and mouth during audiovisual speech segmentation. *Front. Psychol.* 7:52. 10.3389/fpsyg.2016.00052 26869959 PMC4735377

[B30] McGurk H. MacDonald J. (1976). Hearing lips and seeing voices. *Nature* 264:746. 10.1038/264746a0 1012311

[B31] Moray N. (1959). Attention in dichotic listening: affective cues and the influence of instructions. *Q. J. Exp Psychol.* 11 56–60. 10.1080/17470215908416289

[B32] Parasov Z. Chai J. (2008). “What’s in a gaze? The role of eye-gaze in reference resolution in multimodal conversation interfaces,” in *IUI ’08: Proceedings of the 13th International Conference on Intelligent User Interfaces*, (Gran Canaria: Association for Computing Machinery), 1–10.

[B33] Pichora-Fuller M. K. (2003). Processing speed and timing in aging adults: psychoacousitics, speech perception, and comprehension. *Int. J. Audiol.* 42 59–67. 10.3109/14992020309074625 12918611

[B34] Pomper U. Chait M. (2017). The impact on visual gaze direction on auditory object tracking. *Sci. Rep.* 7 1–16. 10.1038/s41598-017-04475-1 28680049 PMC5498632

[B35] Posner M. I. (1980). Orienting of attention. *Q. J. Exp. Psychol.* 32 3–25. 7367577 10.1080/00335558008248231

[B36] Riggio L. Kirsner K. (1997). The relationship between central cues and peripheral cues in covert visual orientation. *Percept. Psychophys.* 59 885–899. 10.3758/bf03205506 9270363

[B37] Rosenblum L. D. Johnson J. A. SaldanÞa H. M. (1996). Point-light facial displays enhance comprehension of speech in noise. *J. Speech Lang. Hear. Res.* 39 1159–1170. 10.1044/jshr.3906.1159 8959601

[B38] Stuart A. Butler A. K. (2014). No learning effect observed for reception thresholds for sentences in noise. *Am. J. Audiol.* 23 227–231. 10.1044/2014_AJA-14-0005 24700076

[B39] Sumby W. H. Pollack I. (1954). Visual contribution to speech intelligibility in noise. *J. Acoust. Soc. Am.* 26 212–215. 10.1121/1.1907309

[B40] Taitelbaum-Swead R. Fostick L. (2016). The effect of age and type of noise on speech perception under conditions of changing context and noise levels. *Folia Phoniatr. Logop.* 68 16–21. 10.1159/000444749 27362521

[B41] Tas A. C. Luck S. J. Hollingworth A. (2016). The relationship between visual attention and visual working memory encoding: a dissociation between covert and overt orienting. *J. Exp. Psychol. Hum. Percept. Perform.* 42:1121. 10.1037/xhp0000212 26854532 PMC4977214

[B42] Tharpe A. M. Ashmead D. Sladen D. P. Ryan H. A. Rothpletz A. M. (2008). Visual attention and hearing loss: past and current perspectives. *J. Am. Acad. Audiol.* 19 741–747. 10.3766/jaaa.19.10.2 19358454

[B43] Thomas S. M. Jordan T. R. (2004). Contributions of oral and extraoral facial movement to visual and audiovisual speech perception. *J. Exp. Psychol. Hum. Percept. Perform.* 30 873–888. 10.1037/0096-1523.30.5.873 15462626

[B44] Tiippana K. Andersen T. S. Sams M. (2004). Visual attention modulates audiovisual speech perception. *Eur. J. Cogn. Psychol.* 16 457–472. 10.1080/09541440340000268

[B45] Treisman A. Squire R. Green J. (1974). Semantic processing in dichotic listening? A replication. *Mem. Cogn.* 2 641–646. 10.3758/bf03198133 24203732

[B46] Treisman A. M. (1960). Contextual cues in selective listening. *Q. J. Exp. Psychol.* 12 242–248. 10.1080/17470216008416732

[B47] Tye-Murray N. Sommers M. S. Spehar B. (2007). Audiovisual integration and lipreading abilities of older adults with normal and impaired hearing. *Ear Hear.* 28 656–668. 10.1097/aud.0b013e31812f7185 17804980

[B48] Vatikiotis-Bateson E. Eigsti I. M. Yano S. Munhall K. G. (1998). Eye movement of perceivers during audiovisualspeech perception. *Percept. Psychophys.* 60 926–940. 10.3758/bf03211929 9718953

[B49] Wendt D. Kollmeier B. Brand T. (2015). How hearing impairment affects sentence comprehension: using eye fixations to investigate the duration of speech processing. *Trends Hear.* 19 1–18. 10.1177/2331216515584149 25910503 PMC4409940

[B50] Yamasoba T. Lin F. R. Someya S. Kashio A. Sakamoto T. Kondo K. (2013). Current concepts in age-related hearing loss: epidemiology and mechanistic pathways. *Hear. Res.* 303 30–38. 10.1016/j.heares.2013.01.021 23422312 PMC3723756

